# The Crystal Structure of Dodecahedral Ba^2+^ Hexa-Perchlorate Complex Tetrakis 1-N-Propyl-3-vinyl-imidazol-1-ium·Barium Hexa-Perchlorate

**DOI:** 10.3390/molecules29215010

**Published:** 2024-10-23

**Authors:** Yuval Zertal, Natalia Fridman, Levi Gottlieb, Yoav Eichen

**Affiliations:** 1 Schulich Faculty of Chemistry, Technion−Israel Institute of Technology Technion City, Haifa 3200003, Israel; 2 RAFAEL, Advanced Defense Systems Ltd., POB 2250, Haifa 3102101, Israel

**Keywords:** energetic materials, ionic liquids, high coordination number

## Abstract

In cold methanol, energetic ionic liquid 1-n-propyl-3-vinyl-imidazol-1-ium perchlorate, **1**, crystallizes in the presence of excess Ba(ClO_4_)_2_, **2**, into tetrakis 1-propyl-3-vinyl-imidazol-1-ium·barium hexa-perchlorate, **3**. Crystals of **3**, with molecular formula (C_8_H_13_N_2_)_4_·BaCl_6_O_24_, are colorless and monoclinic, with space group P2_1_/c. The crystal structure is characterized by a dodecahedral coordination around the barium atom, with each perchlorate chelating Ba^2+^ in a κ^2^O,O’ fashion, and the Ba(ClO_4_)_6_^4−^ anion is surrounded by four imidazolium cations.

## 1. Introduction

Numerous 1,3-dialkyl Imidazolium perchlorates and nitrates, as well as 1-alkyl-3-vinyl Imidazolium perchlorate and nitrate derivatives, have been shown to be room-temperature energetic ionic liquids (**RTEILs**) [1,2,3,4,5]. Recently, they have been suggested as monomers for effective fuel binders in propellant compositions that are also suitable for additive manufacturing of propellant grains [6,7,8,9,10]. The major advantage of this family of materials is that they contain both a cationic organic fuel and an anionic oxidizer in their molecular structure, thereby facilitating the initial steps of combustion and reducing the overall oxygen balance of the fuel part [11,12]. Despite their highly energetic nature, many of these materials fulfill the requirements for the safe handling of energetic materials in terms of shock and electrostatic initiation and are thermally stable up to temperatures exceeding 200 °C [13,14]. 

**Scheme 1:**
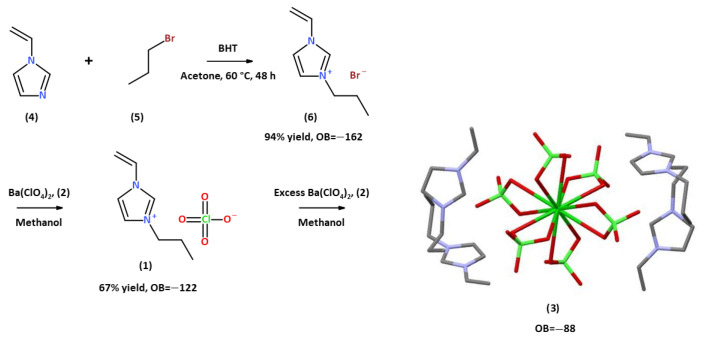
Synthesis of 1-n-propyl-3-vinyl Imidazolium perchlorate, **1**, and preparation of tetrakis 1-propyl-3-vinyl-imidazol-1-ium·barium hexa-perchlorate, **3**.

**Figure 1:**
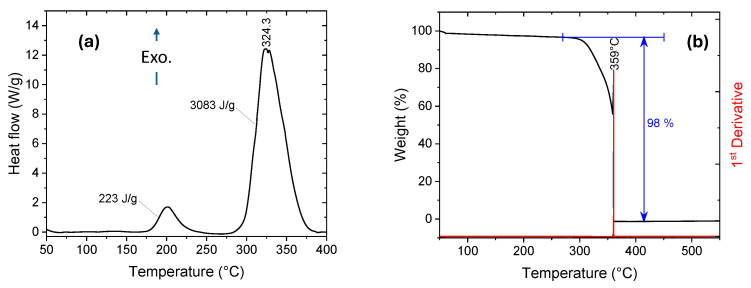
(**a**) DSC and (**b**) TGA curves of 1-n-propyl-3-vinyl Imidazolium perchlorate, **1**. Scan rate = 10 °C·min^−1^.

Yet another advantage of these materials is that the synthesis of 1-alkyl-3-vinyl Imidazolium perchlorates and nitrates is a simple, high-yield process, which is a significant advantage when preparing ingredients for propellants and explosives. For example, the synthesis of 1-n-propyl-3-vinyl Imidazolium perchlorate, **1**, occurs through a two-step procedure from readily available and inexpensive materials; see Scheme 1. Ion metathesis using a solution of barium perchlorate, Ba(ClO_4_)_2_, **2**, in methanol yields a room-temperature energetic ionic liquid (**RTEIL**), which is thermally stable up to ~175 °C. At around 200 °C, the material undergoes a thermally induced polymerization process, followed by a highly exothermic thermal decomposition of the resulting polymer with an onset at ~300 °C and a peak at 324 °C, ΔH_d_ = 3083 J·g^−1^, which is >60% larger than the decomposition enthalpy of RDX measured on the same machine under the same conditions and calibration; see Figure 1a. TGA measurements reveal that the mass loss peaks sharply at 359 °C and reaches a constant level of 98% right after the weight loss peak; see Figure 1b.

Interestingly, the addition of excess Ba(ClO_4_)_2_, **2**, to solutions of **1** in cold methanol resulted in formation of a colorless crystalline material, which was identified as tetrakis 1-propyl-3-vinyl-imidazol-1-ium barium hexa-perchlorate, **3**, (C_8_H_13_N_2_)_4_·BaCl_6_O_24_, characterized by an oxygen balance of OB = −88%, not far from the oxygen balance of TNT, OB = −74%. This involves the coordination of four perchlorate anions of four units of three to the barium atom of Ba(ClO_4_)_2_, forming a distorted dodecahedral coordination around it. At room temperature, the crystals melt and form a liquid phase.

In the past, a systematic study has been conducted on the crystal coordination of the barium ion in various compounds whose structures have been solved. It revealed that, although Ba^2+^ should normally exhibit a coordination number of nine or ten, one can find a variety of coordination modes with coordination numbers varying from six to twelve [15]. Some of the factors that might possibly explain this wide range of coordination numbers were discussed.

## 2. Results

### 2.1. Crystal Structure Determination of Tetrakis 1-N-Propyl-3-vinyl-imidazol-1-ium Barium Hexa-Perchlorate, ***3***

**Figure 2:**
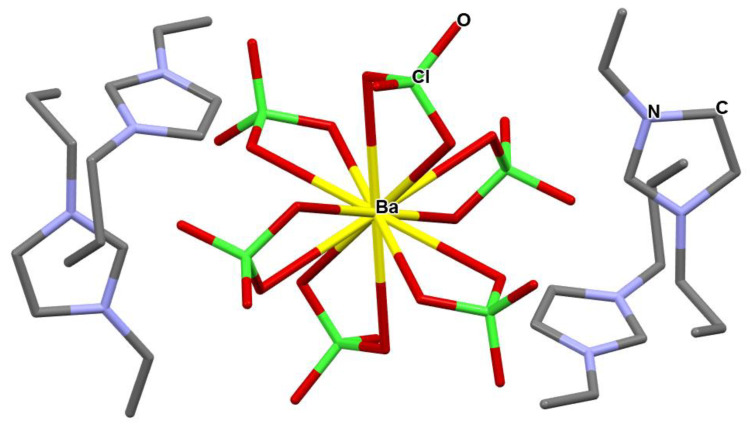
Crystal structure of tetrakis 1-n-propyl-3-vinyl-imidazol-1-ium barium hexa-perchlorate, **3**.

The crystal structure of **3** and its structural information are presented in Figure 2 and in Table 1.

### 2.2. Supramolecular Features

**Figure 3:**
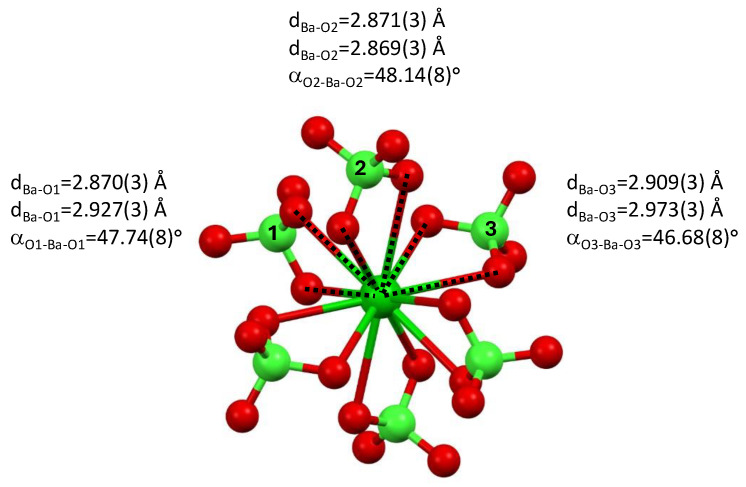
Bonding of the perchlorate anions to the barium cation in tetrakis 1-n-propyl-3-vinyl-imidazol-1-ium barium hexa-perchlorate, **3**.

The perchlorate anions are bound to the barium cation through three almost equivalent, yet different, binding modes; see Figure 3. Two sets of perchlorate molecules, related to one another through an inversion center, are characterized by Ba-O bonds of d_Ba1-O_ = 2.870(3) Å and d_Ba-O1_ = 2.927(3) Å and O-Ba-O angle of α_O1-Ba-O1′_ = 47.74(8)°. Two sets of perchlorate anions, related to one another through an inversion center, are characterized by Ba-O bonds of d_Ba-O2_ = 2.871(3) Å and d_Ba-O2_ = 2.869(3) Å and O-Ba-O angle of α_O2-Ba-O2′_ = 48.12(8)°. Two sets of perchlorate molecules, related to one another through an inversion center, are characterized by Ba-O bonds of d_Ba-O3_ = 2.909(3) Å and d_Ba-O3_ = 2.973(3) Å and O-Ba-O angle of α_O3-Ba-O3′_ = 46.68(8)°.

**Figure 4:**
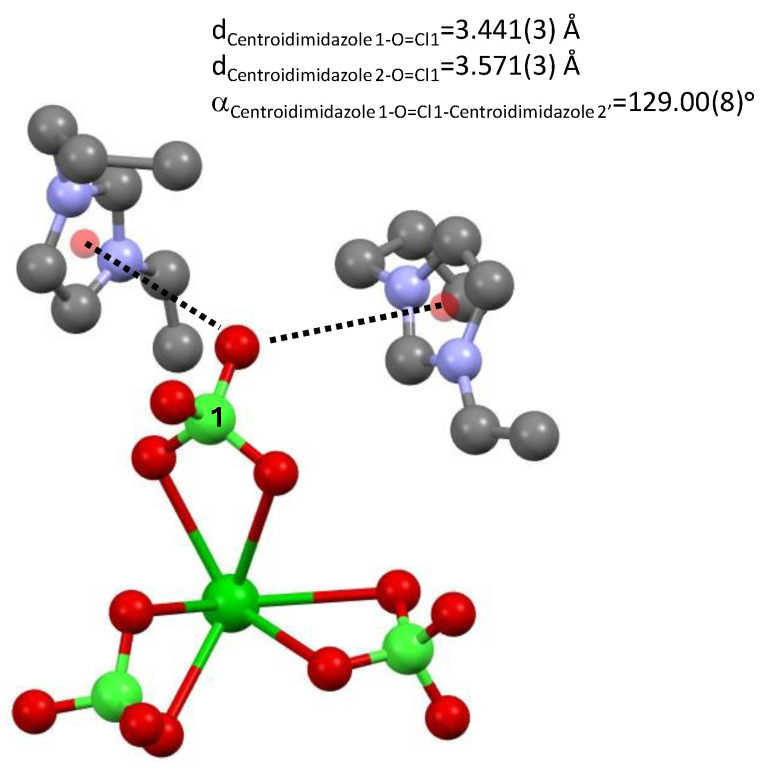
Distances between an oxygen atom of perchlorate **1** and two imidazolium rings of tetrakis 1-n-propyl-3-vinyl-imidazol-1-ium barium hexa-perchlorate, **3**.

An oxygen atom of perchlorate **1** (and its symmetry equivalent) interacts with two π clouds of two imidazolium rings d_Centroidimidazole1-O=Cl1_ = 3.441(3) Å and d_Centroidimidazole2-O=Cl1_ = 3.571(3) Å, α _Centroidimidazole1-O=Cl1-Centroidimidazole2′_ = 129.00(8)°; see Figure 4.

## 3. Refinement

The single crystal of colorless plate material **3** was immersed in Paratone–N oil and mounted on a Rigaku Oxford Diffraction—XtaLAB Synergy-S at 100 K. Data collection was performed using monochromated Mo Kα radiation, λ = 0.71073 Å, using φ and ω scans to cover the Ewald sphere. Accurate cell parameters were obtained with the amount of indicated reflections. Using Olex2 [16], the structure was solved with the olex2.solve [17] structure solution program using charge flipping and refined with the ShelXL [18] refinement package using least squares minimization. All non-hydrogen atoms were refined with anisotropic displacement parameters. The hydrogen atoms were refined isotropically on calculated positions using a riding model with their *U*_iso_ values constrained to 1.5 times the *U*_eq_ of their pivot atoms for terminal sp^3^ carbon atoms and 1.2 times for all other carbon atoms. Software used for molecular graphics: Mercury 2022.3.0 [19].

## 4. Materials and Methods

All starting materials reported in the manuscript were purchased from Sigma-Aldrich (St. Louis, MO, USA). Solvents and starting materials were used as received unless otherwise noted.

NMR spectra were recorded on a Bruker-AVIII-400 spectrometer (Bremen, Germany). Liquid chromatography high-resolution mass spectrometry measurements were performed on Thermo Scientific UltiMate 3000 HPLC (Boston, MA, USA) using 70:30 Acetonitrile:H_2_O as solvent and a flow of 0.5 mL/min, with Brucker maxis impact HRMS ESI. Differential scanning calorimetry, DSC, measurements were conducted on a Q100 TA instrument, using aluminum crucibles with perforated covers. DSC curves were taken under an N_2_ atmosphere at 10 °C min^−1^ from ambient to 400 °C. Thermo-gravimetric analysis, TGA, measurements were conducted on a Q50 TA instrument (New Castle, DE, USA), using open aluminum crucibles. TGA curves were taken under an N_2_ atmosphere at 10 °C min^−1^ from ambient to 500 °C. 

### 4.1. Synthesis of 1-N-Propyl-3-vinyl-imidazol-1-ium Bromide (***6***)

Vinyl imidazole, **4** (20.00 g, 212.52 mmol), and 2,6-di-tert-butyl-4-methylfenol (BHT) (0.0023 g, 0.0106 mmol, 100 ppm) were added to a 100 mL round bottom flask. The mixture was stirred for 5 min, and then, 1- bromopropane, **5** (33.18 g, 233.77 mmol), was added, and the mixture was heated and kept at 60 °C for 3 days, after which a phase separation occurred.

Excess 1- bromopropane was removed under reduced pressure, and the crude product was dissolved in methanol, placed in a separating funnel, and washed with hexane (3 × 50 mL). The methanol phase was collected, and 100 ppm of BHT was added before the solvent was removed under reduced pressure, affording the product (43.43 g, 200.04 mmol) in the form of a light yellow, thick oil, in 94% yield.

^1^H NMR (400 MHz, DMSO-*d*_6_, δ): 9.69 (d, *J* = 1.7 Hz, 1H, Ar H), 8.28 (d, *J* = 1.5 Hz, 1H, Ar H), 7.99 (d, *J* = 2.4 Hz, 1H, Ar H), 7.35 (dd, *J* = 15.6, 8.7 Hz, 1H, C**H**=CH_2_), 6.01 (dd, *J* = 15.7, 2.4 Hz, 1H, CH=C**H**_2_), 5.43 (dd, *J* = 8.8, 2.3 Hz, 1H, CH=C**H**_2_), 4.20 (t, *J* = 7.1 Hz, 2H, N-C**H**_2_-CH_2_), 1.85 (h, *J* = 7.3 Hz, 2H, -CH_2_-C**H**_2_-CH_3_), 0.88 (t, *J* = 7.3 Hz, 3H, -CH_2_-C**H**_3_); ^13^C NMR (101 MHz, DMSO-*d*_6_, δ): 135.81 (Ar C), 129.35 (Ar C), 123.74 (Ar C), 119.68 (**C**H=CH_2_), 109.13 (CH=**C**H_2_), 51.14 (N-**C**H_2_-CH_2_-), 23.06 (-CH_2_-**C**H_2_-CH_3_), 10.91 (-CH_2_-CH_2_-**C**H_3_); HRMS (ESI) *m*/*z*: [M]^+^ calcd. for C_8_H_13_N_2_: 137.1073; found: 137.1082. See Supplementary Materials.

### 4.2. Synthesis of 1-N-Propyl-3-vinyl-imidazol-1-ium Perchlorate (***1***)

**6** (43.43 g, 200.04 mmol) was dissolved in 100 mL of methanol. Barium perchlorate, **2** (33.63 g, 100.02 mmol), was added to a separate beaker and dissolved in 120 mL of methanol. The solution of **6** was added to the beaker containing the barium perchlorate solution, and white precipitate formed. The mixture was diluted with 200 mL of methanol and stirred for 5 min before it was transferred to polypropylene tubes and centrifuged (4000 RPM, 15 min).

The liquid was filtered through a sintered glass Buchner (pore size: 10–16 μm), and the solvent was removed under reduced pressure, affording the product, **1** (31.66 g, 133.78 mmol), in the form of a light yellow, thick oil, in 67% yield.

^1^H NMR (400 MHz, DMSO) δ 9.42 (s, 1H), 8.16 (t, *J* = 1.8 Hz, 1H), 7.88 (t, *J* = 1.6 Hz, 1H), 7.26 (dd, *J* = 15.6, 8.8 Hz, 1H), 5.93 (dd, *J* = 15.6, 2.4 Hz, 1H), 5.42 (dd, *J* = 8.7, 2.4 Hz, 1H), 4.16 (t, *J* = 7.2 Hz, 2H), 1.92–1.75 (m, 2H), 0.88 (t, *J* = 7.4 Hz, 3H); ^13^C NMR (101 MHz, DMSO) δ 135.24, 128.82, 123.26, 119.26, 108.87, 51.00, 22.71, 10.44; HRMS (ESI) m/z: [M]^+^ calcd. for C_8_H_13_N_2_: 137.1073; found: 137.1081. See Supplementary Materials.

### 4.3. Growth of Crystals of ***3***

A sample of **1** containing excess Ba(ClO_4_)_2_, **2**, was kept at −18 °C in a freezer. After 24 h, solid was observed at the bottom of the vial. The liquid was decanted, and the crystalline solid was inspected under a light microscope, to select a crystal that was suitable for structure determination, using single-crystal x-ray diffraction.

## Figures and Tables

**Table 1:** Structural information of the crystal of **3**.

Crystal Data	3
Empirical formula	(C_8_H_13_N_2_)_4_·BaCl_6_O_24_
Formula weight	1282.85
Temperature (K)	100.15
Wavelength (Å)	0.71073
Crystal system	Monoclinic
Space group	P2_1_/c
a (Å)	19.6704(6)
b (Å)	13.4428(4)
c (Å)	20.1021(6)
α (°)	90
β (°)	100.218(3)
γ (°)	90
Volume (Å^3^)	5231.2(3)
Z	4
Calculated density (mg/cm^3^)	1.629
Absorption coefficient (mm^−1^)	1.151
F(000)	2600
Crystal size (mm)	0.15 × 0.15 × 0.06
2Theta range	4.946–60.168
Reflection collected/unique	41,869/11,966
R_int_	0.0649
Completeness (%)	99.6
Absorption correction	semi-empirical
Data/restraints/parameters	11,966/678/711
Goodness-of-fit on F^2	1.011
R1, wR2 [I > 2sigma(I)]	0.0469, 0.0868
R1, wR2 (all data)	0.0919, 0.1003
Largest diff. peak and hole	1.08/−0.58
Diffractometer	XtaLAB Synergy-S

## Data Availability

CCDC 2384212 contains the supplementary crystallographic data for this paper. These data can be obtained free of charge from The Cambridge Crystallographic Data Centre via www.ccdc.cam.ac.uk/data_request/cif (accessed on 1 October 2024).

## Supplementary Materials

The following supporting information can be downloaded at: https://www.mdpi.com/article/10.3390/molecules29215010/s1, Figure S1: ^1^H NMR spectrum of **6**. Figure S2: ^13^C NMR spectrum of **6**. Figure S3: ^1^H NMR spectrum of **1**. Figure S4: ^13^C NMR spectrum of **1**.
