# Supplementary material for: The Crystal Structure of Dodecahedral Ba2+ Hexa-Perchlorate Complex Tetrakis 1-N-Propyl-3-vinyl-imidazol-1-ium·Barium Hexa-Perchlorate
Source: Molecules. 2024 Oct 23;29(21):5010. doi: 10.3390/molecules29215010 (PMC11547198; doi:10.3390/molecules29215010)
Supplement: Supplementary file 1 [file molecules-29-05010-s001.zip › Supporting information.pdf]

Supporting information

The crystal structure of dodecahedral Ba^{2+} hexa-perchlorate complex tetrakis 1-n-propyl-3-vinyl-imidazol-1-ium · barium hexa-perchlorate

Yuval Zertal [1], Natalia Fridman [1], Levi Gottlieb [2,*] and Yoav Eichen [1,*]

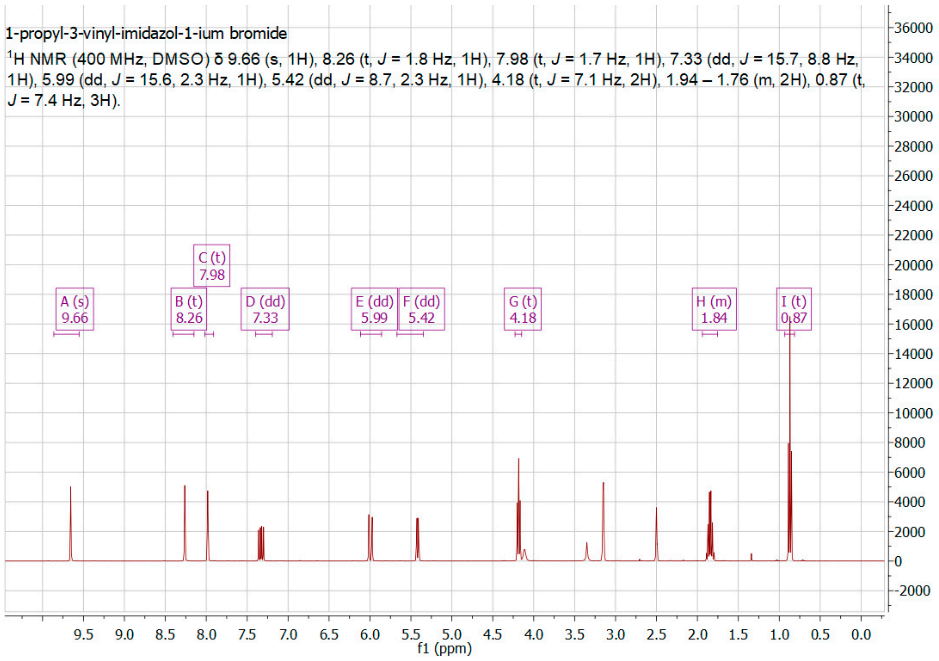

Figure S1: ^{1}H NMR spectrum of **6**.

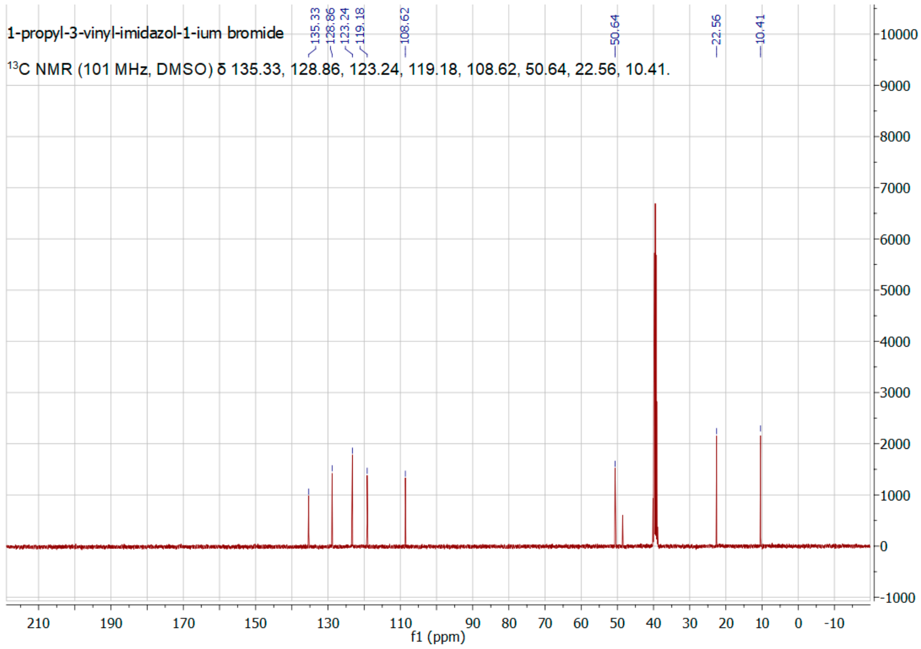

Figure S2: ^{13}C NMR spectrum of **6**.

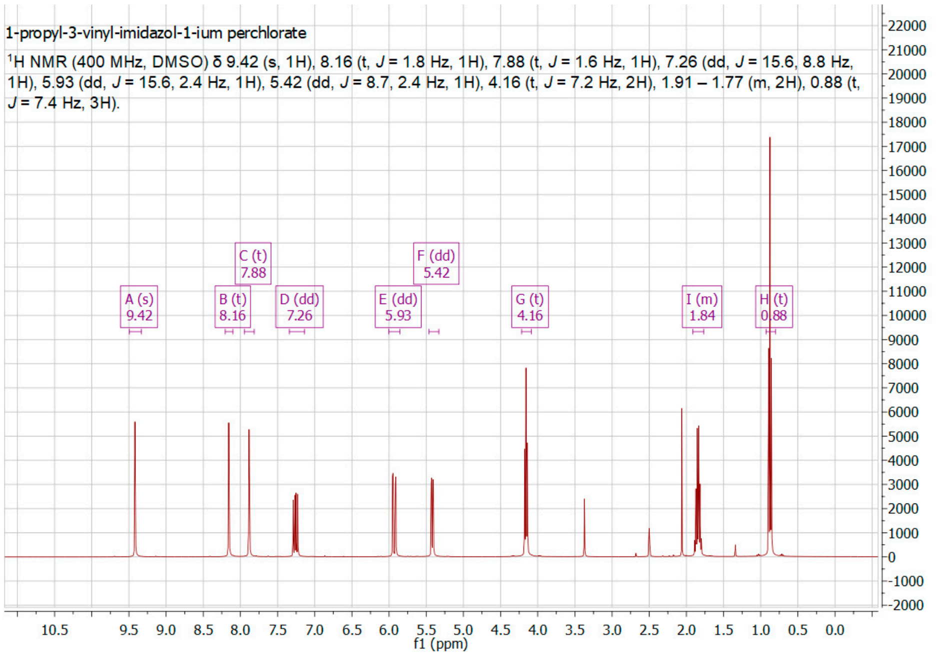

Figure S3: ^{1}H NMR spectrum of **1**.

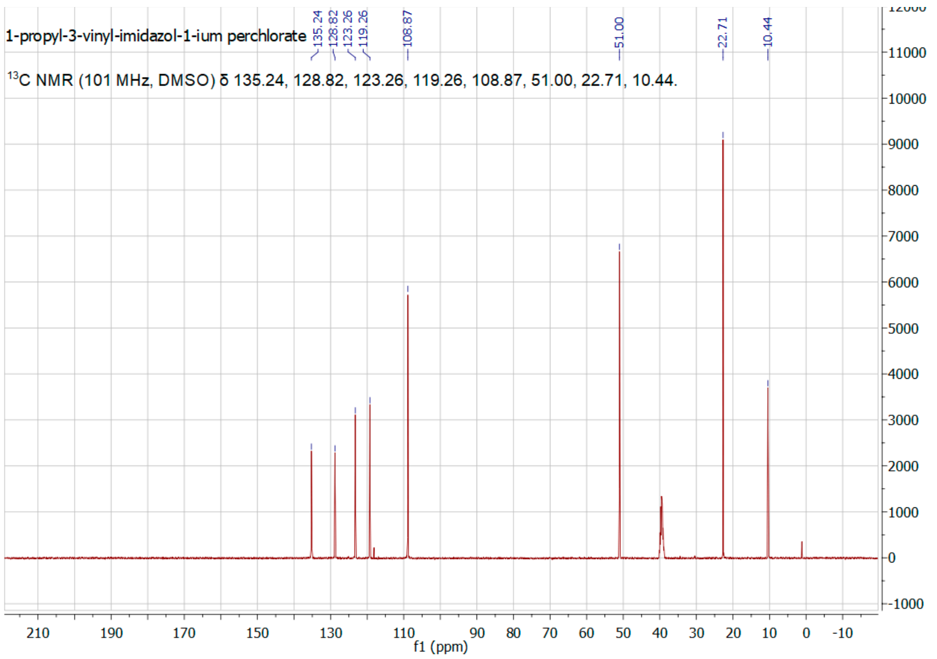

Figure S4: ^{13}C NMR spectrum of **1**.

The oxygen balance of a molecule with the formula $C_aH_bN_cO_dCl_e$, was calculated using Equation S*1*.

Equation S1: Oxygen balance calculation

$$\%OB = \frac{-1600\left(2 \times a + \frac{(b-e)}{2} - d\right)}{Mw}$$

Example: Oxygen balance calculation for **1**:

Molecular formula of **1**, $C_8H_{13}N_2{\cdot}ClO_4$:

$$\%OB = \frac{-1600\left(2 \times 8 + \frac{(13-1)}{2} - 4\right)}{236.6} = -122\%$$
